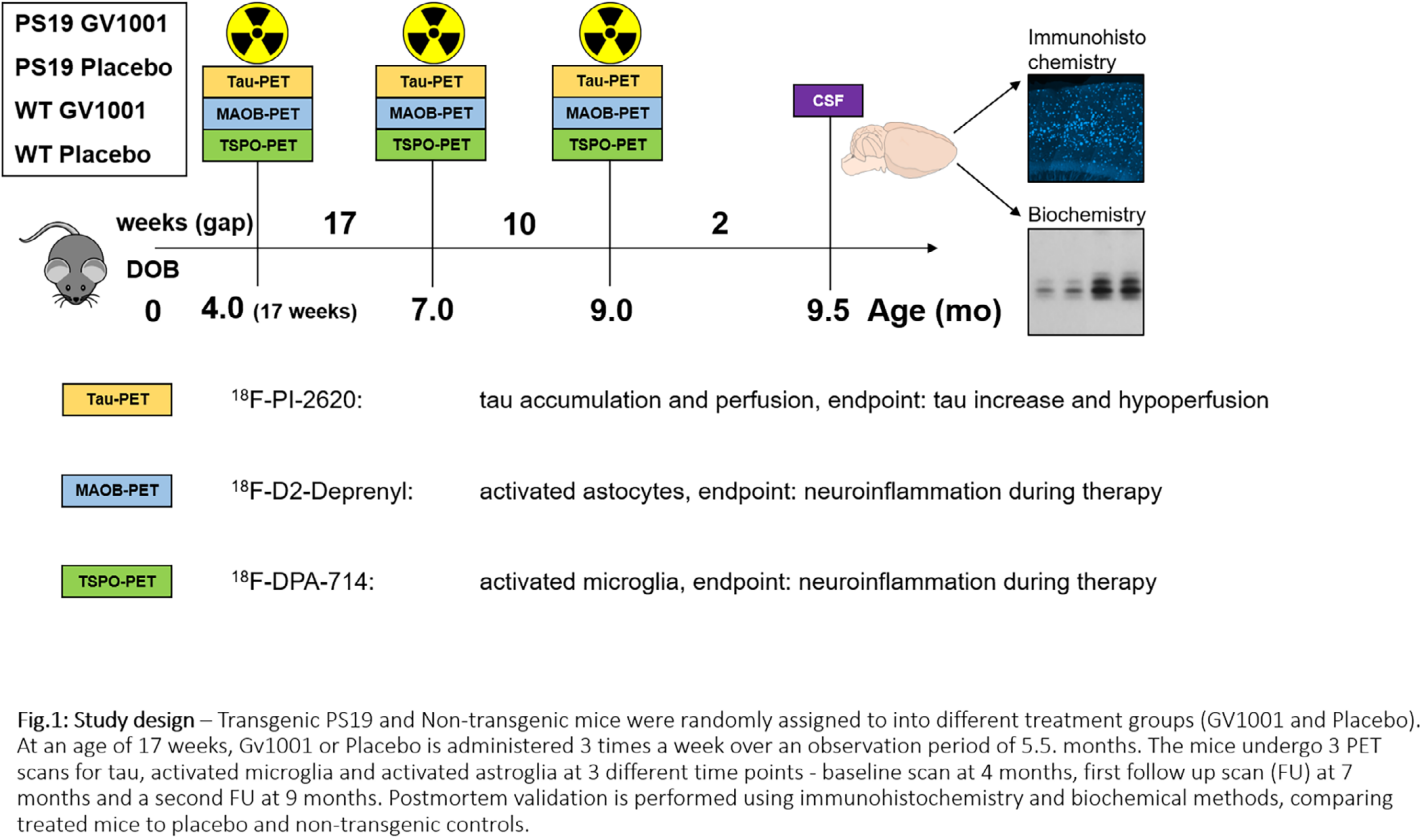# Multi‐tracer PET monitoring of an immunomodulatory therapy in 4R tauopathy: Evaluating a novel drug's impact on glial function and protein pathology

**DOI:** 10.1002/alz70856_104796

**Published:** 2026-01-07

**Authors:** Tim Bathe, Svetlana Salomasova, Manvir Lalia, Lea Helena Kunze, Giovanna Palumbo, Rosel Oos, Emanuel Joseph, Matthias Brendel

**Affiliations:** ^1^ Ludwig‐Maximilians‐University Munich / LMU University Hospital, Munich, Bavaria, Germany; ^2^ Graduate School of Systemic Neurosciences, Munich, Bavaria, Germany; ^3^ Ludwig‐Maximilians‐University Munich, Munich, Bavaria, Germany; ^4^ LMU University Hospital, Munich, Bavaria, Germany; ^5^ Institute for Stroke and Dementia Research (ISD), University Hospital, LMU, Munich, Bavaria, Germany; ^6^ University Hospital, Ludwig‐Maximilians‐Universität, Munich, Germany; ^7^ German Center for Neurodegenerative Diseases (DZNE), Munich, Germany; ^8^ Munich Cluster for Systems Neurology (SyNergy), Munich, Bavaria, Germany

## Abstract

**Background:**

The prevalence of neurodegenerative diseases (ND), including Alzheimer's disease (AD) and non‐AD tauopathies, is projected to rise significantly by 2050 due to an aging global population. Chronic neuroinflammation, driven by glial activation in response to protein pathologies, is a major contributor to disease progression. Targeting glial dysfunction through immunomodulatory therapies offers a promising approach to mitigate the effects of tauopathies and other ND.

**Method:**

PS19 mice receive chronic treatment with GV1001 over 5 months. Serial neuroimaging techniques, including PET scans targeting tau protein, microglial activation, and astrocytic responses, are employed to assess treatment effects in vivo (Figure 1). Postmortem validation is performed using immunohistochemistry and biochemical methods, comparing treated mice to placebo and non‐transgenic controls.

**Result:**

The research scope is to monitor the efficacy of GV1001 in a transgenic tau mouse model (PS19) with an early‐intervention biomarker study using molecular biology and neuroimaging techniques including TSPO (microglia) PET, deprenyl (astroglia) PET, tau PET (perfusion and retention) and CSF markers of inflammation (e.g. sTREM2) and neurodegeneration (NfL).

Preliminary findings, expected to be presented at the conference, will provide insights into the drug's ability to modulate glial activity, restore homeostasis, and reduce tau pathology.

**Conclusion:**

This study highlights the potential of monitoring immunomodulatory strategies to address the complex interplay between chronic neuroinflammation and protein aggregation in ND. If successful, these findings could inform the development of novel therapeutic approaches for AD and related disorders, bridging the gap between preclinical research and clinical application.